# *Lactobacillus reuteri* mitigates hepatic ischemia/reperfusion injury by modulating gut microbiota and metabolism through the Nrf2/HO-1 signaling

**DOI:** 10.1186/s13062-024-00462-5

**Published:** 2024-03-18

**Authors:** Leiyi Zhang, Xiaoxiang Gong, Juan Tan, Rongsen Zhang, Mingxia Li, Cong Liu, Chenhao Wu, Xiaojing Li

**Affiliations:** 1 Department of General Surgery, The Second Xiangya Hospital, Central South University, No. 139 Renmin Middle Road, Furong District, 410011 Changsha, China; 2 Department of Pediatrics, The Second Xiangya Hospital, Central South University, 410011 Changsha, China; 3 Research Associate Department of Pathology, The Xiangya Third Hospital, Central South University, 410013 Changsha, China; 4 Department of Ultrasound Diagnosis, The Second Xiangya Hospital, Central South University, 410011 Changsha, China; 5 Department of Anesthesiology, Wuhan Third Hospital, Tongren Hospital of Wuhan University, 430061 Wuhan, China

**Keywords:** Hepatic ischemia/reperfusion injury, *Lactobacillus reuteri*, Gut microbiota, Metabolism, Nrf2/HO-1 pathway

## Abstract

**Background:**

This study seeks to investigate the impacts of *Lactobacillus reuteri* (*L. reuteri*) on hepatic ischemia-reperfusion (I/R) injury and uncover the mechanisms involved.

**Methods:**

Mice in the I/R groups were orally administered low and high doses of *L.reuteri* (*L.reuteri*-low and *L. reuteri*-hi; 1 × 10^10^ CFU/d and 1 × 10^11^ CFU/d), for 4 weeks prior to surgery. Following this, mice in the model group were treated with an Nrf2 inhibitor (ML-385), palmitoylcarnitine, or a combination of both.

**Results:**

After treatment with *L. reuteri*, mice exhibited reduced levels of serum aminotransferase (ALT), aspartate aminotransferase (AST), and myeloperoxidase (MPO) activity, as well as a lower Suzuki score and apoptosis rate. *L. reuteri* effectively reversed the I/R-induced decrease in Bcl2 expression, and the significant increases in the levels of Bax, cleaved-Caspase3, p-p65/p65, p-IκB/IκB, p-p38/p38, p-JNK/JNK, and p-ERK/ERK. Furthermore, the administration of *L. reuteri* markedly reduced the inflammatory response and oxidative stress triggered by I/R. This treatment also facilitated the activation of the Nrf2/HO-1 pathway. *L. reuteri* effectively counteracted the decrease in levels of beneficial gut microbiota species (such as *Blautia*, *Lachnospiraceae NK4A136*, and *Muribaculum*) and metabolites (including palmitoylcarnitine) induced by I/R. Likewise, the introduction of exogenous palmitoylcarnitine demonstrated a beneficial impact in mitigating hepatic injury induced by I/R. However, when ML-385 was administered prior to palmitoylcarnitine treatment, the previously observed effects were reversed.

**Conclusion:**

*L. reuteri* exerts protective effects against I/R-induced hepatic injury, and its mechanism may be related to the promotion of probiotic enrichment, differential metabolite homeostasis, and the Nrf2/HO-1 pathway, laying the foundation for future clinical applications.

**Supplementary Information:**

The online version contains supplementary material available at 10.1186/s13062-024-00462-5.

## Introduction

Hepatic ischemia/reperfusion (I/R) injury is a significant complication commonly seen in hepatic transplantation, resection, and cases of hemorrhagic shock [1]. This type of injury occurs when there is an insufficient oxygen supply to the liver, followed by reperfusion, leading to inflammatory responses and oxidative stress that result in cell death [2]. Although pharmacotherapy has shown promise in preventing or mitigating I/R injury in experimental settings, there is still a lack of effective strategies and validated pharmacological targets for clinical application. This is due to a limited understanding of the complex signaling events triggered by I/R injury, as well as challenges such as short circulation time, poor solubility, and severe side effects associated with available treatments [3].

The gut microbiota and its metabolites play a crucial role in regulating the development of hepatic I/R injury [4, 5]. 3,4-dihydroxyphenylpropionic acid attenuates hepatic I/R injury via regulating inflammatory activity [5]. Metabolic reprogramming promoted by glutamine originating from gut microbiota helps decrease hepatic I/R injury [6]. Probiotics can alter the host’s microbial community and provide beneficial health effects. *Lactobacillus reuteri* (*L. reuteri*) has been recognized as a probiotic for improving hepatic health, and dietary *L. reuteri* prevents lipopolysaccharide (LPS)-mediated hepatocyte apoptosis by improving gut flora and bile acid metabolism [7, 8]. *L. reuteri DSM 17938* improves gut microbiota to reduce d-galactosamine-induced hepatic failure in rats [9]. *L. reuteri* can reduce ischemic injury-induced cardiac damage [10]. Clostridium butyricum supplements prevent hepatic I/R injury by modulating gut microbial composition, which helps to reduce LPS and attenuate inflammation and oxidative stress [11]. The role of *L. reuteri* in hepatic I/R injury is currently unclear.

The development of hepatic I/R injury is largely due to oxidative stress and an exaggerated inflammatory response [12]. The Nrf2/HO-1 axis is a multi-organ protective chain against oxidative stress damage [13]. Senkyunolide I attenuates hepatic I/R injury in mice through the Nrf2/HO-1 pathway [14]. Curculigoside attenuates hepatic I/R injury-induced oxidative stress, inflammation, and apoptosis by activating the Nrf2/HO-1 pathway [15]. *APS1* (a kind of *Lactobacillus mali*) ameliorates hepatic steatosis by modulating lipid metabolism through in vivo regulation of specific non-alcoholic fatty liver disease-associated gut microbiota [16]. However, the precise impacts of *L. reuteri* on hepatic I/R injury remain unclear. In this study, we investigated the effects and mechanisms of *L. reuteri* on hepatic I/R injury by constructing a mouse model with or without *L. reuteri* pretreatment. Our findings offer a novel approach to probiotic therapy for treating hepatic I/R injury.

## Materials and methods

### Culture and preparation of freeze-dried powder of *L. reuteri*

The bacterium *L. reuteri* was cultured under anaerobic conditions for 18 h in a sterile environment using the De Man Rogosa and Sharpe medium at a temperature of 37 °C. As soon as the culture reached the logarithmic growth phase, it was harvested and subsequently centrifuged at a rate of 4000×g for 5 min, maintained at 4 °C. The bacterial strain was then suspended in skimmed milk and freeze-dried to create a powder-like form using a vacuum over 14 h. The resultant strain powder was securely stored in sealed packaging at a temperature of 4 °C. The potency of the strain powder was quantified through the plate count method. *L. reuteri* was dissolved in phosphate buffered saline (PBS) for use.

### Animal treatment

The study was approved by the Animal Ethical and Welfare Committee, The Second Xiangya Hospital, CSU (NO.20230479). C57BL/6J mice (8 ~ 10 weeks, males) were purchased from Hunan SJA Laboratory Animal Co., Ltd (China). All mice were housed in specific pathogen-free animal facilities and acclimatized for one week before starting the experiment. Hepatic I/R models were constructed based on previously described methods [5, 17]. Specifically, after being anesthetized, the blood supply to the left hepatic lobe and the middle lobe of the hepatic of mice was blocked by an arterial clip. Mice were ischemic for 90 min, after which the clamps were removed. Then, mice were reperfused for 1, 6, and 24 h. *L. reuteri* was purchased from Abiowell (Changsha, China). Mice in the Sham and I/R groups were orally administered vehicle (PBS), and low and high doses of *L.reuteri* (1 × 10^10^ CFU/d, *L.reuteri*-low; 1 × 10^11^ CFU/d, *L. reuteri*-hi), for 4 weeks before surgery [8]. Mice were euthanized at reperfusion for 1, 6, and 24 h, respectively. Figure S1 (top) illustrates the details of the experiment.

To further investigate the effect of the metabolite palmitoylcarnitine on hepatic I/R injury, mice were further divided into 5 groups, including Sham, I/R, ML-385, palmitoylcarnitine, and ML-385 + palmitoylcarnitine groups. The treatment steps for the I/R group were as described previously. Briefly, mice in the I/R group were reperfused for 6 h after being ischemic for 90 min. Palmitoylcarnitine (1 µM) was added to the perfusate at the beginning of reperfusion in both the palmitoylcarnitine group and ML-385 + palmitoylcarnitine group mice [18]. Mice in the Sham group underwent the same surgical procedures except for vascular occlusion. Mice in the ML-385 group and the ML-385 + palmitoylcarnitine group were injected with ML-385 (Nrf2 inhibitor, 30 mg/kg, 30 min before surgery) by intraperitoneal injection, respectively, prior to surgery [15]. Mice were euthanized after 6 h of reperfusion. Blood, hepatic, and colonic feces were collected. Since the time of surgery was closely related to the severity of hepatic injury, we completed the first surgery in mice between 8 and 9 a.m. In addition, mice were fasted for 1 h before surgery. Figure S1 (down) illustrates the details of the experiment.

### Biochemical analysis and enzyme-linked immunosorbent assay (ELISA)

Serum alanine aminotransferase (ALT), aspartate aminotransferase (AST), and myeloperoxidase (MPO), as well as superoxide dismutase (SOD), malondialdehyde (MDA), glutathione (GSH), and glutathione peroxidase (GPx) activity levels were determined using biochemical kits (Nanjing Jiancheng Institute of Bioengineering, China) according to the manufacturer’s instructions. The biochemical kit item numbers for ALT, AST, MPO, SOD, MDA, GSH, and GPx were C009-2-1, C010-2-1, A044-1-1, A001-3-2, A003-1-2, A006-2-1, and A005-1-2, respectively.

Serum IL-1β, IL-6, TNF-α, and IL-10 concentrations were assessed by ELISA assay kits (CUSABIO, China). The ELISA kits for IL-1β, IL-6, TNF-α, and IL-10 were available under the item numbers CSB-E08054m, CSB-E04639m, CSB-E04594m, and CSB-E04741m, respectively.

Finally, a multifunctional enzyme labeling analyzer (MB-530, HEALES, China) was used to measure the optical density (OD_450 nm_) value.

### Hematoxylin and eosin (H&E) staining and Suzuki score

As described previously [5], H&E was used to stain paraffin-embedded hepatic tissues. A light microscope (BA210T, Motic, China) was used to visualize the H&E-stained sections. As mentioned earlier [5], following the Suzuki scoring system, the histological damage score was calculated by adding up the scores for three distinct parameters: congestion, vacuolization, and necrosis. The scoring system assigns a range of 0 to 4 for each parameter, with a maximum score of 12.

### TdT-mediated dUTP nick end labeling (TUNEL)

TUNEL kit (40306ES50, Shanghai Yeasen Biologicals) was used for fluorescence staining of paraffin sections. A fluorescence microscope (BA410T, Motic, China) was used to observe the sections after staining.

### Western blot

In this process, we initially extracted the entire protein content. Subsequently, we utilized SDS-PAGE to separate the proteins, followed by transfer onto nitrocellulose membranes. Next, nitrocellulose membranes were incubated with primary antibodies (shown in Table S1) at 4 °C overnight. After that, nitrocellulose membranes were incubated with secondary antibodies (anti-mouse/rabbit IgG) for 90 min. A gel imaging system imaged protein bands after color development/exposure (ChemiScope6100, CLINX, China). PCNA (nucleus) and β-actin were internal references.

### Quantitative real-time PCR (qRT-PCR)

Total cellular RNA was extracted and subsequently reverse-transcribed. Primer sequences for the Nrf2, HO-1, and β-actin genes are shown below. Nrf2 (143 bp), forward, 5’-GCTCCTATGCGTGAATCCCAA-3’, reverse, 5’-TTTGCCCTAAGCTCATCTCGT-3’; HO-1 (191 bp), forward, 5’-TCCATGTTGACTGACCACGACT-3’, reverse, 5’-CCCACCCCTCAAAAGATAGCC-3’; β-actin (223 bp), forward, 5’-ACATCCGTAAAGACCTCTATGCC-3’; reverse, 5’-TACTCCTGCTTGCTGATCCAC-3’. Relative expression of target genes was calculated using the 2^−ΔΔCt^ method with β-actin as an internal reference, and each sample was analyzed in triplicate.

### 16S rRNA sequencing

Fecal DNA extraction was performed using the DNeasy PowerSoil Pro kit (Qiagen, USA). The V3 to V4 variable regions of the 16S rRNA gene were amplified by PCR using universal primers (341F: 5’-CCTACGGGGNGGCWGCAG-3’; 805R: 5’-GACTACHVGGGTATCTAATCC-3’). After amplification, purification and characterization, sequencing was performed on the Illumina NovaSeq6000 platform to construct gene libraries. Finally, representative reads were selected using the Qiime 2 software package. A linear discriminant analysis of effect sizes (LefSe) was used with LDA to identify feature taxa.

### Immunofluorescent (IF) staining

IF staining was performed as described previously [19]. After being treated with heat-repair antigen and other routine treatments, the sections and primary antibodies (ZO-1, claudin-3, and occludin) (shown in Table S2) were incubated at 4 °C overnight. After that, the sections were incubated with the secondary antibody (anti-rabbit IgG(H + L)) at 37 °C for 90 min. Fluorescence microscopy was used to observe the expression of the target protein.

### Liquid chromatography-mass spectrometry (LC-MS) analysis

The LC-MS analysis was conducted using an Agilent 1290 Infinity ultra-high performance liquid chromatography system coupled with an Agilent 6545 UHD and Accurate-Mass Q-TOF mass spectrometer. A Waters XSelect R HSS T3 column (2.5 μm, 100 × 2.1 mm) was utilized for chromatographic separation. The mobile phases comprised 0.1% formic acid in water solution (A) and 0.1% in acetonitrile (B). The flow rate and column temperature were set at 0.4 mL/min and 40 °C, respectively. The chromatographic gradient program involved the following steps: 0–3 min, 20% B; 3–9 min, 20–95% B; 9–13 min, 95% B; 13-13.1 min, 95 − 5% B; and 13.1–16 min, 5% B. Mass spectra were acquired in the range of 50-1500 m/z. Agilent Masshunter Qualitative Analysis B.08.00 software (Agilent Technologies, USA) and R software were employed for data analysis.

### Statistical analysis

Statistical analysis was performed using Prism software (version 8.0). The data were presented as means ± standard deviation (SD). We used the Student’s t-test to analyze differences between the two groups, while the one-way analysis of variance (ANOVA) was implemented for comparisons across multiple groups. The level of significance was set at ^*^P < 0.05.

## Results

### *L. reuteri* attenuates hepatic I/R injury

**Fig. 1:**
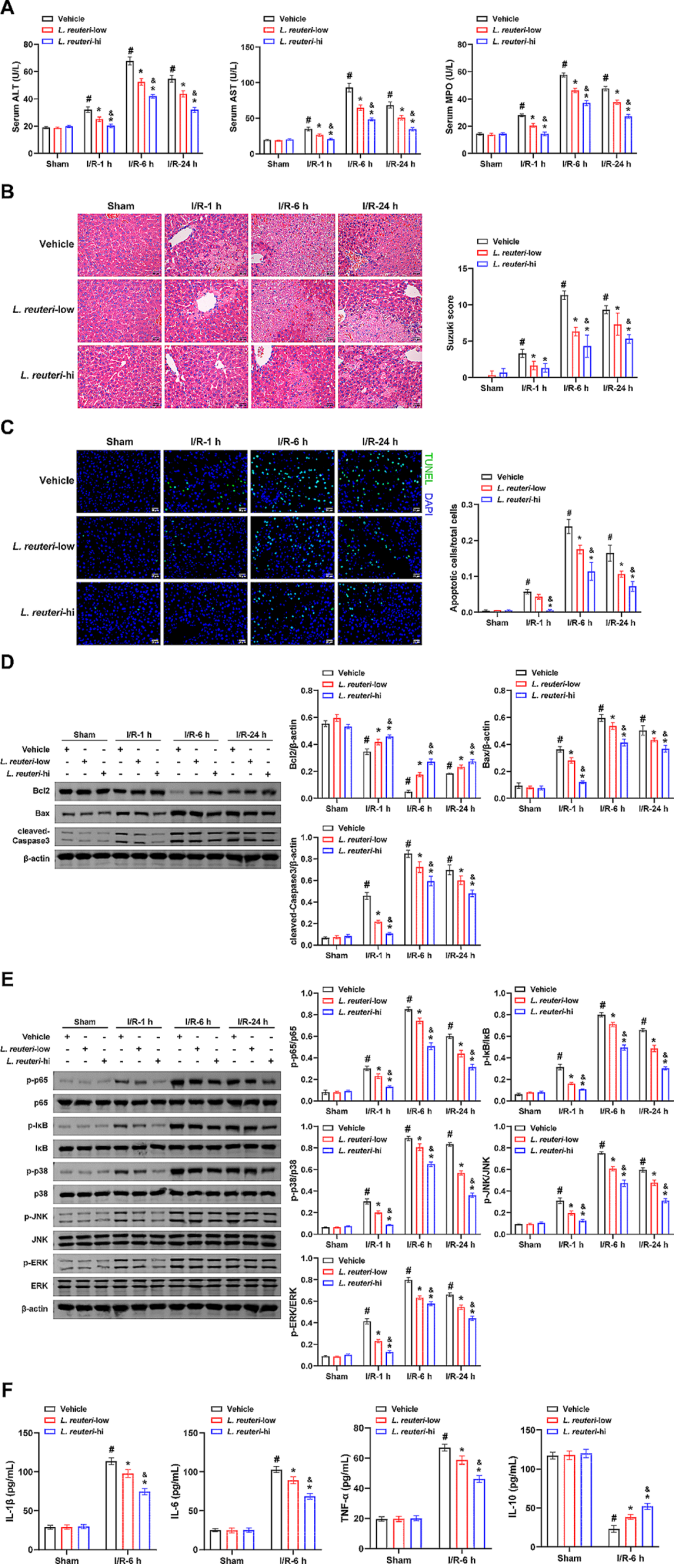
*L. reuteri* inhibits hepatic I/R-induced tissue damage, apoptosis, and inflammation. **(A)** Serum ALT, AST, and MPO activity levels were measured by biochemical kits. **(B)** The degree of hepatic tissue damage was observed by H&E staining, and Suzuki scores were obtained. Scale bar: 25 μm (400×). **(C)** The level of cell apoptosis in hepatic tissue was assessed by TUNEL fluorescence staining. Scale bar: 25 μm (400×). **(D)** The expression of Bcl2, Bax, and cleaved-Caspase3 was detected by western blot. **(E)** The levels of p-p65/p65, p-IκB/IκB, p-p38/p38, p-JNK/JNK, and p-ERK/ERK were detected by western blot. **(F)** Concentrations of IL-1β, IL-6, TNF-α, and IL-10 were assessed by ELISA. *n* = 10 mice/group. ^#^*P <* 0.05 vs. Sham + vehicle; ^*^P < 0.05 I/R-1 h + vehicle, I/R-6 h + vehicle, or I/R-24 h + vehicle; ^&^*P <* 0.05 I/R-1 h + *L. reuteri*-low, I/R-6 h + *L. reuteri*-low, or I/R-24 h + *L. reuteri*-low

First, mice were utilized as a model to study hepatic I/R injury. The results showed significant increases in serum ALT, AST, and MPO activity levels, Suzuki score, and apoptosis rate in mice affected by I/R injury, with the most pronounced changes observed after 6 h of reperfusion. Subsequently, mice in the experimental group were administered *L. reuteri* treatment. The findings revealed that serum ALT, AST, MPO activity levels, Suzuki score, and apoptosis rate exhibited a concentration-dependent reduction in *L. reuteri*-treated mice, with a more pronounced impact observed in the *L. reuteri*-hi group (Fig. 1A, B, and C). *L. reuteri* administration demonstrated a mitigating effect on I/R-induced hepatic vacuolization and sinusoidal congestion, with a more pronounced impact observed in the *L. reuteri*-hi group (Fig. 1B). Following I/R injury, a significant decrease in Bcl2 expression was observed. Conversely, the Bax and cleaved-Caspase3 expressions were significantly elevated, with the most significant changes in the I/R-6 h group. Notably, *L. reuteri* treatment exhibited a reversal of these indicators, and the effect was more pronounced in the *L. reuteri*-hi group than in the *L. reuteri*-low group (Fig. 1D). The activation of JNK/p38, ERK, and NF-κB pathways has been closely implicated in hepatic I/R injury [20–22]. The ratios of p-p65/p65, p-IκB/IκB, p-p38/p38, p-JNK/JNK, and p-ERK/ERK levels were found to be significantly elevated in the I/R group compared to the Sham group. The peak values of these ratios were observed after 6 h of reperfusion. Conversely, in the presence of *L. reuteri* treatment, the ratios of p-p65/p65, p-IκB/IκB, p-p38/p38, p-JNK/JNK, and p-ERK/ERK were consistently lower in the *L. reuteri*-low and *L. reuteri*-hi groups when compared to the I/R group. Notably, the lowest levels of these indicators were observed in the *L. reuteri*-hi group (Fig. 1E). *L. reuteri* treatment effectively counteracted the I/R-6 h-induced elevation of IL-1β, IL-6, and TNF-α levels, as well as the corresponding decrease in IL-10 concentrations. Notably, these effects were more pronounced in the *L. reuteri*-hi group (Fig. 1F). Moreover, the administration of *L. reuteri* in low or high doses did not significantly affect these indices in the Sham group mice. These findings indicate that *L. reuteri* can suppress hepatic I/R-related tissue injury, apoptosis, inflammation, and the activation of JNK/p38, ERK, and NF-κB pathways.

### *L. reuteri* relieves oxidative stress injury caused by hepatic I/R and activates the Nrf2/HO-1 pathway

**Fig. 2:**
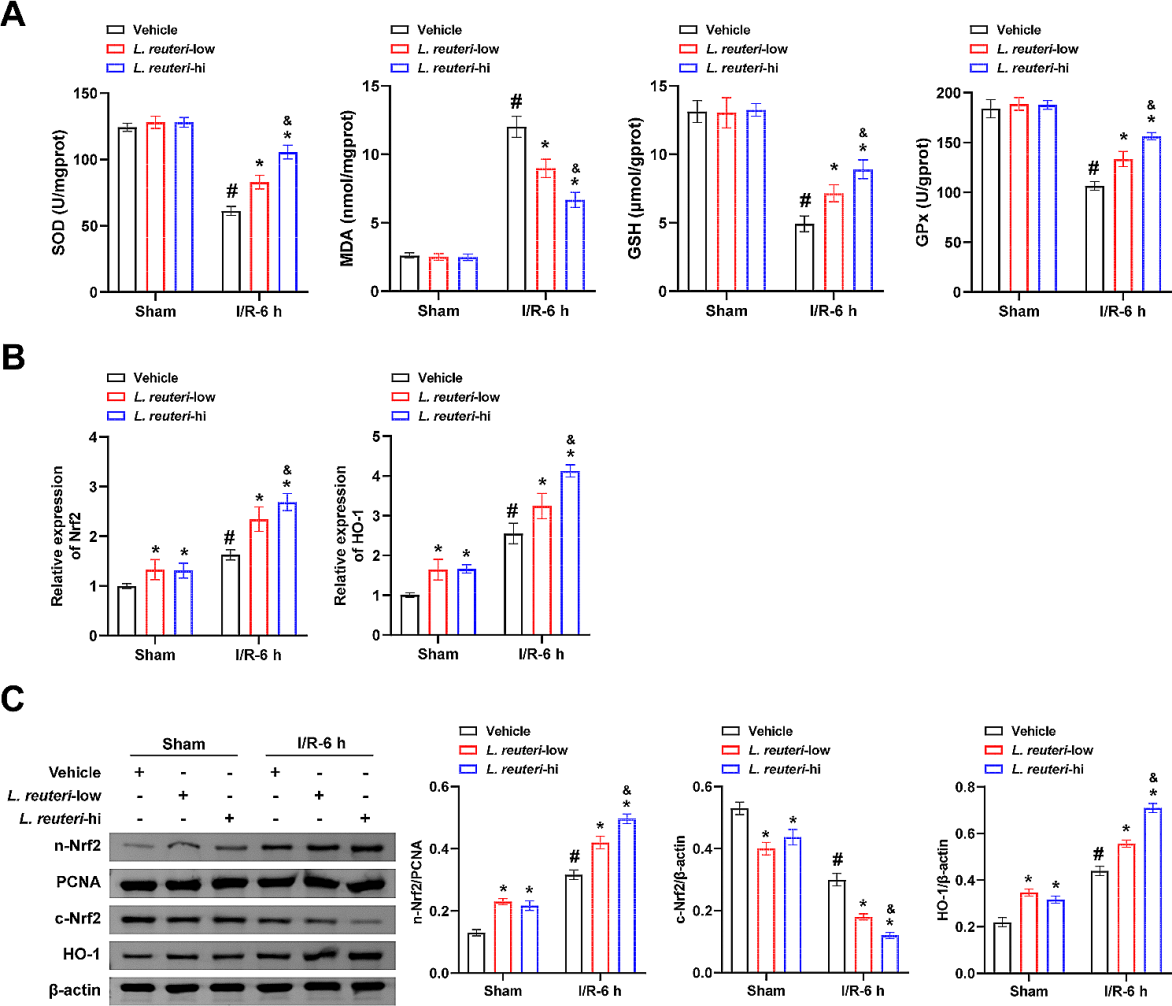
*L. reuteri* alleviates hepatic I/R-induced oxidative stress injury and activates the Nrf2/HO-1 pathway. **(A)** SOD, MDA, GSH, and GPx levels were evaluated by biochemical kits. **(B)** The levels of Nrf2 and HO-1 were determined using qRT-PCR. **(C)** The levels of c-Nrf2, n-Nrf2, and HO-1 were assessed through western blot analysis. *n* = 10 mice/group. ^#^*P <* 0.05 vs. Sham + vehicle; ^*^P < 0.05 vs. I/R-6 h + vehicle. ^&^*P <* 0.05 I/R-6 h + *L. reuteri*-low

To examine the effects of *L. reuteri* on hepatic I/R-induced oxidative stress injury, we measured the levels of SOD, GSH, GPx, and MDA. The data revealed that *L. reuteri* could reverse the I/R-induced decrease in SOD, GSH, and GPx levels while also reducing the elevated MDA levels, with a more pronounced impact observed in the L. reuteri-hi group (Fig. 2A). Notably, the Nrf2/HO-1 axis has been recognized as a vital protective mechanism against oxidative stress, including hepatic I/R injury [13, 14]. *L. reuteri* can enhance intestinal barrier function in rats with d-galactosamine-induced acute hepatic failure through modulation of the Nrf2/HO-1 pathway [23]. Hence, we hypothesized that the beneficial effects of *L. reuteri* on hepatic I/R injury might be associated with the Nrf2/HO-1 pathway. Therefore, we assessed the expression of Nrf2 and HO-1 proteins. Compared to the Sham + vehicle group, the expression of cytoplasmic Nrf2 (c-Nrf2) was significantly reduced in the Sham + *L. reuteri*-low and Sham + *L. reuteri*-hi groups. Conversely, nuclear Nrf2 (n-Nrf2) and HO-1 levels were significantly upregulated. Furthermore, the *L. reuteri*-treated groups exhibited lower levels of c-Nrf2 and higher levels of n-Nrf2 and HO-1 compared to the I/R-6 h + vehicle group. Remarkably, this trend was more pronounced in the *L. reuteri*-hi group, implying that *L. reuteri* facilitates the translocation of Nrf2 into the nucleus, promoting the activation of the Nrf2/HO-1 pathway (Fig. 2B and C). The above results elucidate that the alleviating effect of *L. reuteri* on hepatic I/R-induced oxidative stress injury may be associated with activating the Nrf2/HO-1 pathway.

### *L. reuteri* remodeling gut microbiota in I/R mice

**Fig. 3:**
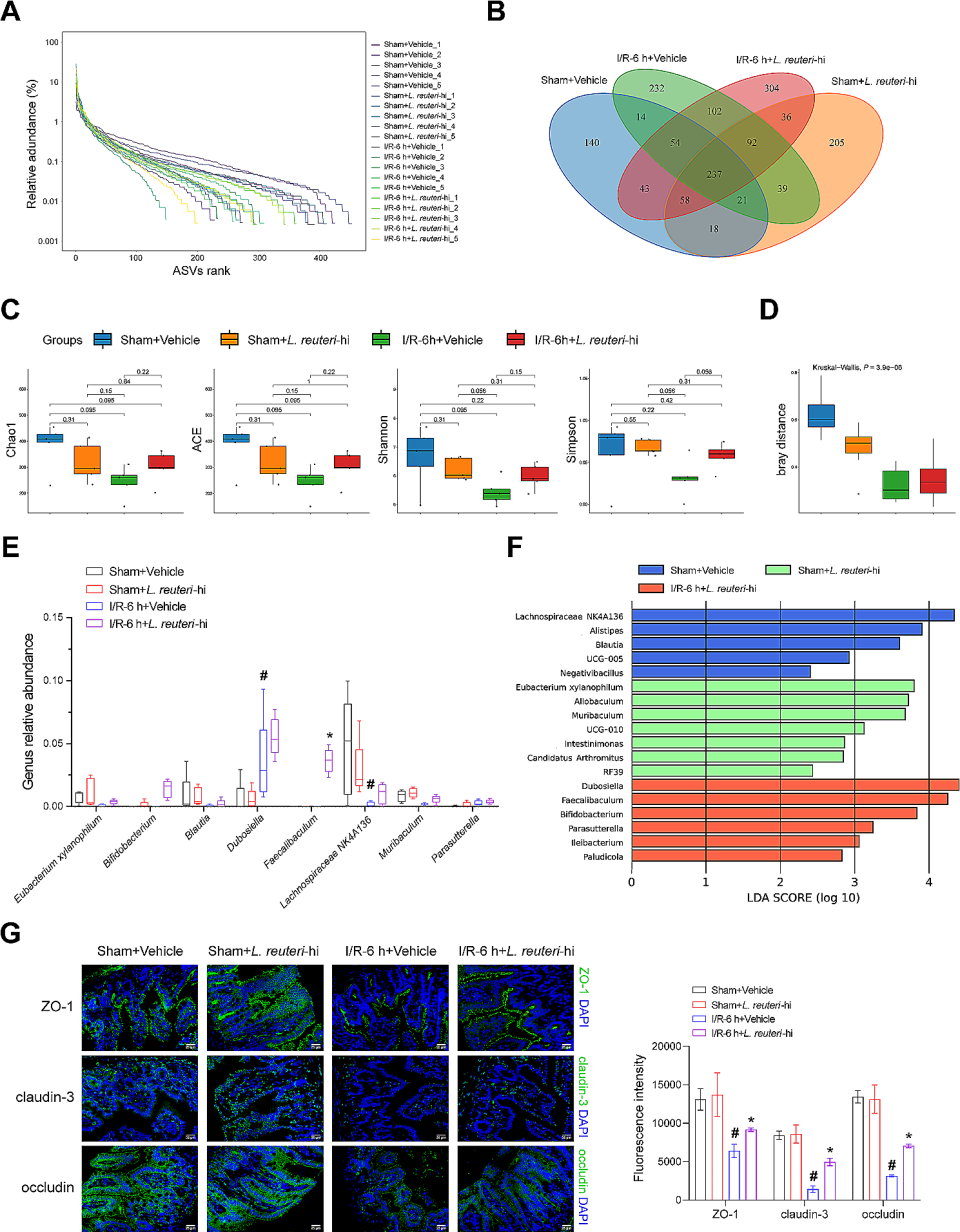
*L. reuteri* alters gut microbiota composition and alleviates intestinal barrier function impairment. **(A)** Rank-abundance curves. **(B)** Venn diagram. **(C)** Alpha diversity analysis including Chao1, ACE, Shannon, and Simpson indices. **(D)** Beta diversity analysis. **(E)** Genus level. **(F)** Lefse. **(G)** The expressions of ZO-1, claudin-3, and occludin were detected by IF staining. Scale bar: 25 μm (400×). *n* = 5 mice/group. ^#^*P <* 0.05 vs. Sham + vehicle; ^*^P < 0.05 vs. I/R-6 h + vehicle.

*L. reuteri* has been shown to protect against LPS-mediated hepatocyte apoptosis through the improvement of gut microbiota [7, 8]. To gain more understanding, we investigated the effect of *L. reuteri* on gut microbiota composition through 16S rRNA sequencing. The rank-abundance curves displayed a satisfactory species abundance and evenness distribution across all groups (Fig. 3A). Venn diagram demonstrating the number of intersecting amplicon sequence variants (ASVs) for the 4 groups was 237 (Fig. 3B). *L. reuteri* treatment counteracted the decreases in Chao1, ACE, Shannon, and Simpson indices induced by I/R injury, indicating that it enhances the number and diversity of microbial species (Fig. 3C). Beta diversity analysis revealed that the dissimilarities between different groups were significantly larger than the differences within each group (Fig. 3D). Furthermore, at the genus level, *L. reuteri* reversed the decline trend in *Eubacterium xylanophilum*, *Blautia*, *Lachnospiraceae NK4A136*, and *Muribaculum* levels induced by I/R injury (Fig. 3E). Lefse analysis demonstrated significant enrichment of *Dubosiella*, *Faecalibaculum*, and *Bifidobacterium* at the genus level in the I/R-6 h + *L. reuteri*-hi group (Fig. 3F). Subsequently, we investigated the impact of *L. reuteri* on hepatic I/R-induced intestinal barrier function. The findings indicated that *L. reuteri* reversed the decrease in the expression of key indicators of intestinal barrier function (ZO-1, claudin-3, and occludin) induced by hepatic I/R injury (Fig. 3G). *L. reuteri* may alleviate hepatic I/R injury by modulating gut microbiota and improving intestinal barrier integrity.

### *L. reuteri* affects hepatic I/R-induced metabolic changes in mice

**Fig. 4:**
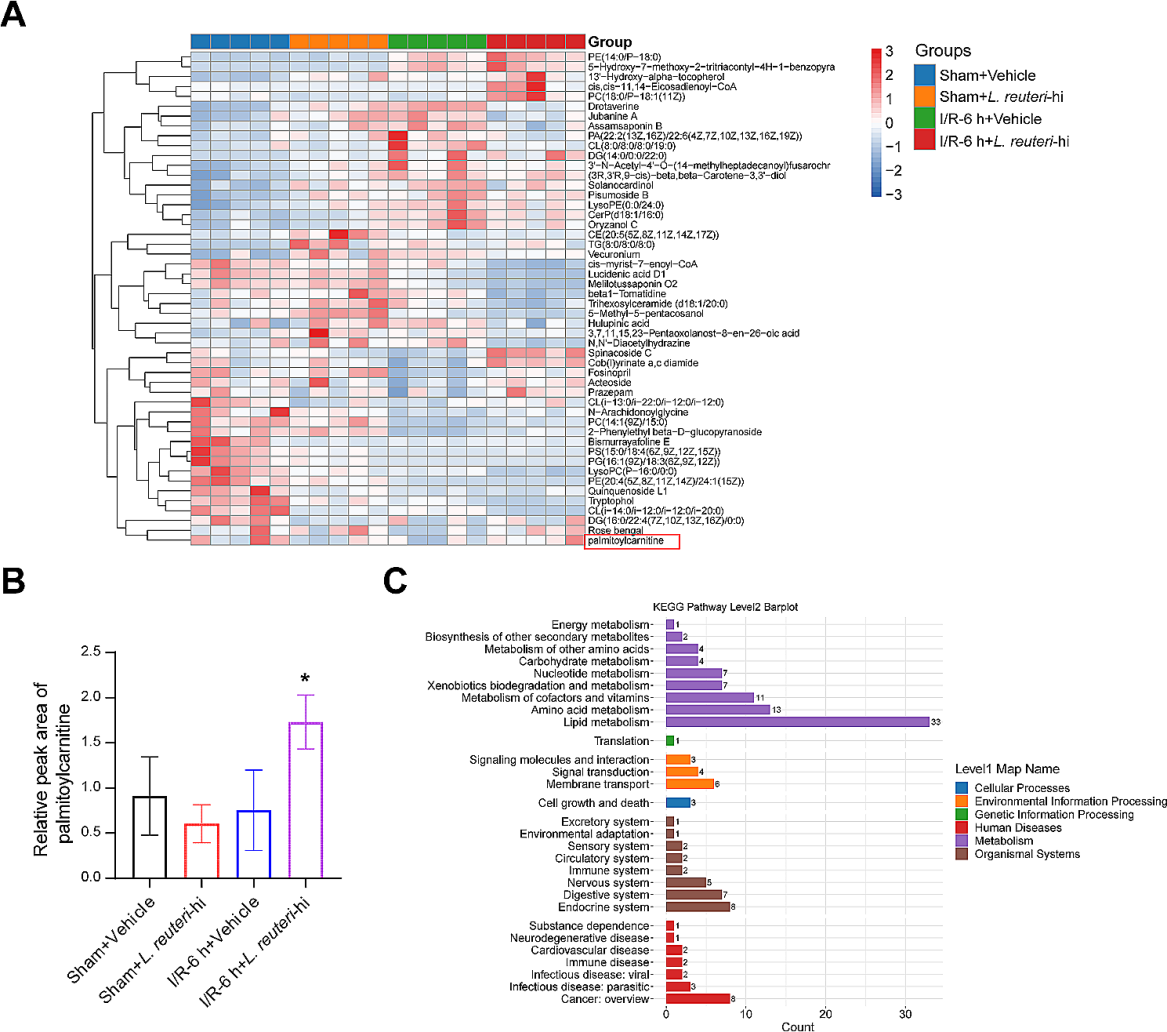
*L. reuteri* partially restores microbial metabolic homeostasis in hepatic I/R-induced mice. **(A)** Heat map of differential metabolites. **(B)** Enrichment of the metabolite palmitoylcarnitine. **(C)** KEGG analysis. *n* = 5 mice/group. ^*^P < 0.05 vs. I/R-6 h + vehicle.

*L. reuteri* has been found to reduce the occurrence of cholestasis-associated microbiota and effectively prevent the inflammatory response and hepatocyte apoptosis in response to acute LPS stimulation [7]. We hypothesized that *L. reuteri* has an impact on hepatic I/R-induced metabolic changes in mice. The administration of *L. reuteri* effectively inhibited the increase in levels of metabolites such as Drotaverine, jubanine A assamsaponin B, PA (22:2(13Z,16Z)/22:6(4Z,7Z,10Z,13Z,16Z,19Z)), and CL (8:0/8:0/8:0/19:0) induced by I/R injury. Additionally, *L. reuteri* reversed the decrease in levels of metabolites, such as Spinacoside C, Cob(I)yrinate a,c diamide, and palmitoylcarnitine caused by I/R injury (Fig. 4A and B). The enriched KEGG pathways observed were lipid metabolism, amino acid metabolism, metabolism of cofactors and vitamins, endocrine system, and cancer overview (Fig. 4C). These findings suggest that *L. reuteri* interventions partially restored microbial metabolic homeostasis in mice with hepatic I/R injury.

### Pearson correlation coefficient analysis

**Fig. 5:**
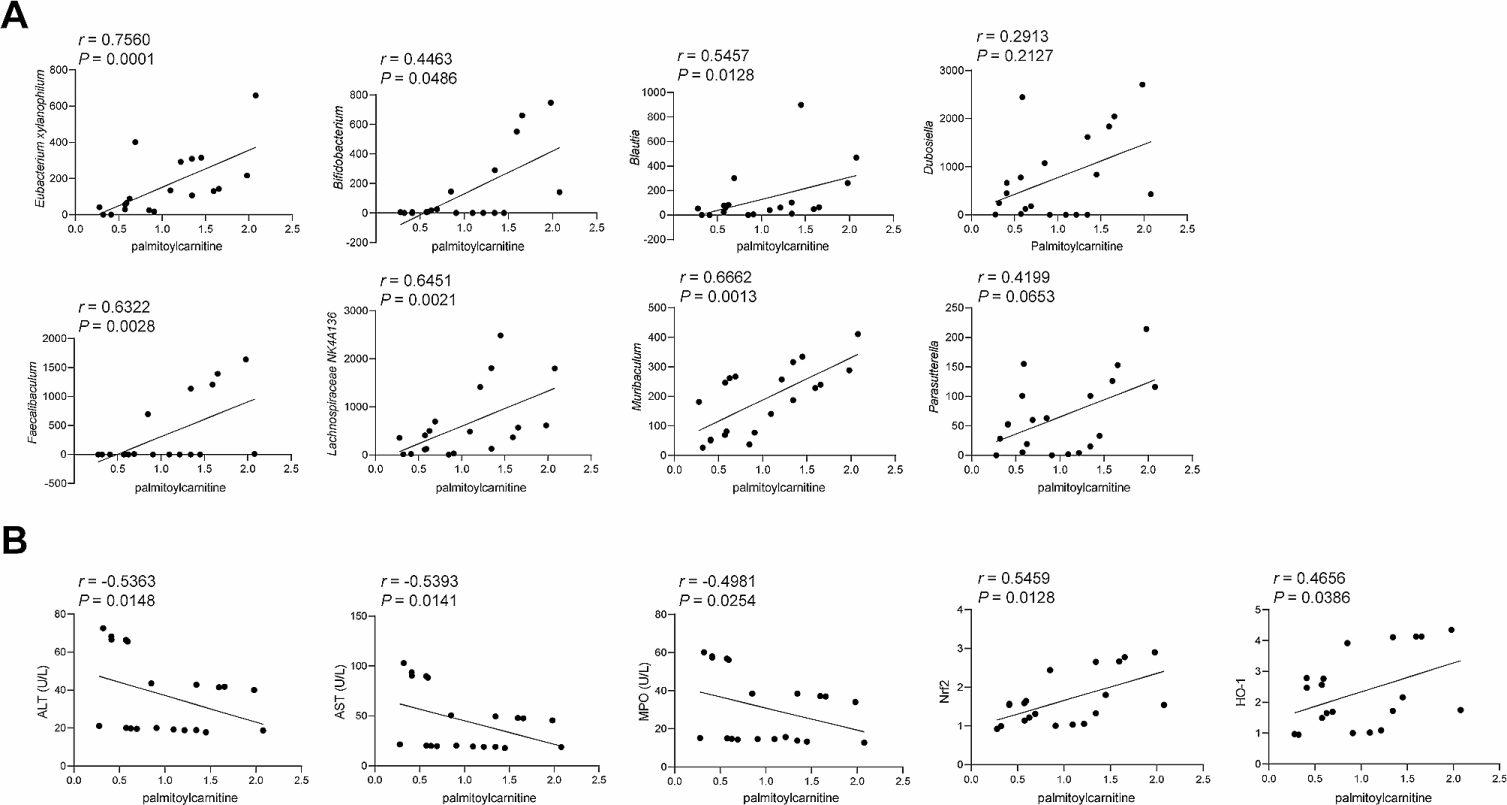
Pearson correlation coefficient analysis. **(A)** Correlation analysis of palmitoylcarnitine with differential gut microbiota. **(B)** Correlation analysis of palmitoylcarnitine and ALT, AST, MPO, Nrf2, and HO-1 levels

We would like to explore further the beneficial metabolites of *L. reuteri* that alleviate hepatic I/R injury. Therefore, the differential metabolites, including spinacoside C, Cob(I)yrinate a,c diamide, and palmitoylcarnitine, were analyzed in correlation with differential gut microbiota, as well as levels of ALT, AST, MPO, Nrf2, and HO-1 using the Pearson correlation coefficient. The enrichment of spinacoside C displayed significant positive correlations with the levels of *Eubacterium xylanophilum*, *Bifidobacterium, Dubosiella, Faecalibaculum*, *Parasutterella*, Nrf2, and HO-1 (Figures S2A-2B). The enrichment of Cob(I)yrinate a,c diamide were significant positive correlations with the levels of *Eubacterium xylanophilum*, *Bifidobacterium, Blautia*, *Dubosiella, Faecalibaculum*, *Muribaculum*, *Parasutterella*, Nrf2, and HO-1 (Figures S3A-3B). However, there was no significant correlation between spinacoside C and Cob(I)yrinate a,c diamide enrichment and the levels of ALT, AST, and MPO (Figures S2B and S3B). The enrichment of palmitoylcarnitine displayed significant positive correlations with the levels of *Eubacterium xylanophilum*, *Bifidobacterium, Blautia*, *Faecalibaculum*, *Lachnospiraceae NK4A136*, *Muribaculum*, Nrf2, and HO-1. Moreover, it exhibited a negative correlation with ALT, AST, and MPO (Fig. 5A and B), demonstrating that palmitoylcarnitine is associated with gut microbiota levels and oxidative stress. The fatty acid-derived mitochondrial substrate palmitoylcarnitine selectively reduces the survival of colorectal and prostate cancer cells [24]. The intermediate fatty acid palmitoylcarnitine confers protection against myocardial I/R injury. Its mechanism of action involves inhibiting complex IV, promoting increased generation of ROS, and activating the RISK pathway [18, 25]. Therefore, we hypothesized a link between palmitoylcarnitine and the protective effects of *L. reuteri* in the liver I/R induction model. Subsequent studies have focused on palmitoylcarnitine.

### Palmitoylcarnitine alleviates hepatic I/R injury through the Nrf2/HO-1 axis

**Fig. 6:**
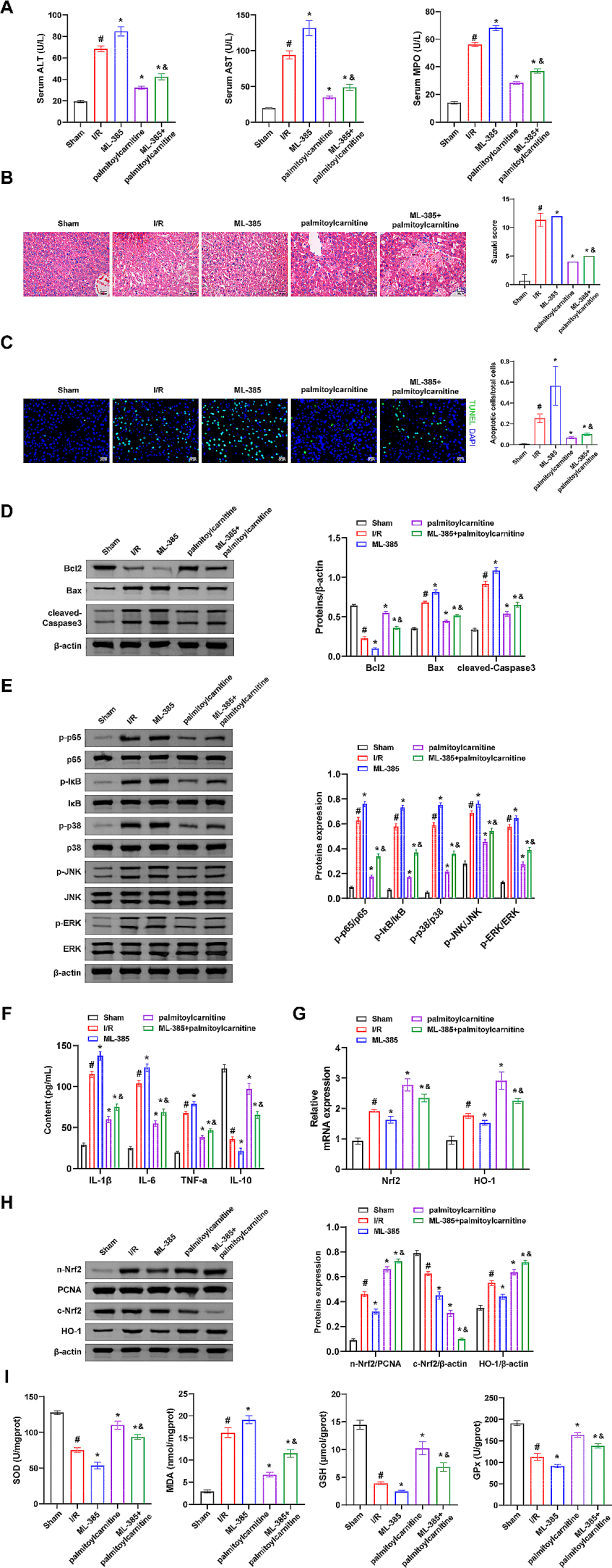
Palmitoylcarnitine alleviates hepatic I/R injury through the Nrf2/HO-1 axis. **(A)** Serum ALT, AST, and MPO activity levels were measured by biochemical kits. **(B)** The degree of hepatic tissue damage was observed by H&E staining, and Suzuki scores were obtained. Scale bar: 25 μm (400×). **(C)** The levels of apoptosis in hepatic tissue were assessed by TUNEL fluorescence staining. Scale bar: 25 μm (400×). **(D)** The expressions of Bcl2, Bax, and cleaved-Caspase3 were detected by western blot. **(E)** The levels of p-p65/p65, p-IκB/IκB, p-p38/p38, p-JNK/JNK, and p-ERK/ERK were detected by western blot. **(F)** IL-1β, IL-6, TNF-α, and IL-10 concentrations were assessed by ELISA. **(G)** The expressions of Nrf2 and HO-1 were detected by qRT-PCR. **(H)** The expressions of c-Nrf2, n-Nrf2, and HO-1 were detected by western blot. **(I)** SOD, MDA, GSH, and GPx levels were evaluated by biochemical kits. *n* = 10 mice/group. ^#^*P <* 0.05 vs. Sham; ^*^P < 0.05 vs. I/R; ^&^*P <* 0.05 vs. palmitoylcarnitine

Next, we investigated whether palmitoylcarnitine mitigates hepatic I/R injury through modulation of the Nrf2/HO-1 axis. Our findings demonstrated that palmitoylcarnitine effectively decreased the serum levels of ALT, AST, and MPO activity induced by I/R injury. Moreover, palmitoylcarnitine reduced the Suzuki score and inhibited the increased trend in apoptotic cell count/total cell count (Fig. 6A, B, and C). Palmitoylcarnitine demonstrated a significant decrease in the levels of Bax, cleaved Caspase3, p-p65/p65, p-IκB/IκB, p-p38/p38, p-JNK/JNK, and p-ERK/ERK induced by I/R injury. Additionally, palmitoylcarnitine significantly increased the levels of Bcl2, which were decreased by I/R injury (Fig. 6D and E). Palmitoylcarnitine effectively reversed the significant increase in IL-1β, IL-6, and TNF-α induced by I/R injury while reversing the significant decrease in IL-10 concentrations (Fig. 6F). Moreover, palmitoylcarnitine activated the expression of n-Nrf2 and HO-1 while inhibiting the expression of c-Nrf2 (Fig. 6G and H). Additionally, palmitoylcarnitine reversed the I/R-induced decrease in levels of SOD, GSH, and GPx, and attenuated the increase in MDA levels (Fig. 6I). Notably, apart from c-Nrf2, the effects of ML-385 on the aforementioned indicators were opposite to those of the palmitoylcarnitine group. Furthermore, the ML-385 + palmitoylcarnitine group effectively reversed the changes in the above indexes observed in the palmitoylcarnitine group. These results reveal that palmitoylcarnitine alleviates hepatic I/R injury by activating the Nrf2/HO-1 axis.

## Discussion

I/R-induced hepatic injury is a key factor affecting the prognosis of hepatic surgery, which can lead to gut microbiota and extrahepatic metabolic disorders [26]. The present study revealed that *L. reuteri* inhibits I/R-induced apoptosis, inflammation, and oxidative stress damage in hepatic tissues. Its mechanism may be related to the promotion of nuclear translocation of Nrf2, the promotion of probiotic enrichment (e.g., *Blautia* and *Lachnospiraceae NK4A136*), and the adjustment of differential metabolite homeostasis (e.g., palmitoylcarnitine). In addition, exogenous palmitoylcarnitine supplementation was similar to the protective effect of *L. reuteri* on the I/R-induced model group, whereas Nrf2 inhibition partially reversed the above effects of palmitoylcarnitine, suggesting that palmitoylcarnitine inhibits I/R through the Nrf2/HO-1 pathway induced hepatic injury.

Disruption of normal gut microbiota is associated with I/R-induced damage to various tissues, such as intestinal and hepatic injury [27, 28]. A variety of probiotics can play a protective role in I/R-induced disease models. For example, *Lactobacillus murinus* mitigates intestinal I/R injury by modulating gut microbiota and macrophage IL-10 release [29]. *VSL#3* ameliorates renal I/R injury by protecting intestinal barrier function, maintaining gut microbiota function, and modulating IL-10/GSK-3β/PTEN pathway-mediated macrophage phenotype [30]. Similarly, Bifidobacterium bifidum BGN4 inhibited I/R-induced renal tissue injury by modulating the colonic microenvironment and gut microbiota [31]. *L. reuteri* attenuated I/R-induced cardiac injury [10]. Similar to the above studies, the current study found that *L. reuteri* alleviated hepatic I/R-induced injury by a mechanism that may be related to the protection of intestinal barrier function and maintenance of gut microbiota homeostasis. *Blautia*, with potential probiotic properties, alleviates LPS-induced acute hepatic injury [32]. *Lactobacillus acidophilus* and *Bifidobacterium* may protect against myocardial ischemia-reperfusion [33]. Our study found that *L. reuteri* treatment increases the enrichment of probiotics (e.g., *Bifidobacterium*, *Blautia*, and *Lachnospiraceae NK4A136*), which might be one of the important reasons for the resistance of *L. reuteri* to hepatic I/R injury.

Metabolites of gut microbiota origin maintain intestinal and systemic homeostasis [34]. *Bifidobacterium breve CCFM1025* attenuates major depression by modulating gut microbiota and tryptophan metabolism [35]. Dietary *L. reuteri*-derived metabolites contribute to immune checkpoint inhibitor therapy in melanoma [36]. Supplementation of *L. reuteri* with the ability to produce Aryl hydrocarbon receptor (AhR) ligands ameliorated E. coli-induced mastitis in an AhR-dependent manner [37]. In this study, *L. reuteri* was found to inhibit the I/R-induced elevation of metabolites drotaverine, jubanine A, assamsaponin B, PA (22:2(13Z,16Z)/22:6(4Z,7Z,10Z,13Z,16Z,19Z)), and CL (8:0/8:0/8:0/19:0). *L. reuteri* reversed the I/R-induced decrease in the levels of the metabolites, such as spinacoside C, cob(I)yrinate a,c diamide, and palmitoylcarnitine. The regulation of hepatic I/R-induced metabolite homeostasis by *L. reuteri* might be important for its protective role. exhibited a negative correlation with the levels of ALT, AST, and MPO. Correlation analysis showed that only palmitoylcarnitine enrichment was negatively correlated with the levels of ALT, AST, and MPO. In addition, considering that spinacoside C and Cob(I)yrinate a,c diamide are less well studied, no commercial products are available for in vivo intervention in animals, and their function in other diseases has not been clarified. Therefore, we have not further explored the effects of Spinacoside C and Cob(I)yrinate a,c diamide on hepatic I/R injury in vivo at this time. The fatty acid-derived mitochondrial substrate palmitoylcarnitine selectively reduces the survival of colorectal and prostate cancer cells [24]. Palmitoylcarnitine protects against myocardial I/R injury, and its mechanisms involving inhibition of complex IV, increased ROS generation, and activation of the RISK pathway [18, 25]. In this study, we found for the first time that palmitoylcarnitine inhibites I/R-induced apoptosis, inflammation, and oxidative stress injury in hepatic tissue.

Up-regulation of downstream antioxidants upon Nrf2 activation can help promote hepatic recovery during I/R [38]. Yuan et al. reported that Gastrodin pretreatment protected the hepatic from I/R injury by activating Nrf2/HO-1 signaling [39]. Consistent with the above studies, the present study found a significant increase in the levels of ALT, AST, MPO, apoptosis, inflammatory factors, and MDA, and a significant decrease in the levels of SOD, GSH, and GPx after ML-385 treatment, exposing that inhibition of Nrf2 promotes I/R-induced hepatic injury. *L. reuteri* alleviates d-galactosamine-induced hepatic injury by promoting the Nrf2/HO-1 axis [23]. Similarly, *L. reuteri* and palmitoylcarnitine promote nuclear translocation of Nrf2, which further activates the downstream HO-1 pathway. *L. reuteri* and palmitoylcarnitine inhibited LPS-induced significant increases in the levels of ALT, AST, MPO, apoptosis, inflammatory factors, and MDA, and significant decreases in the levels of SOD, GSH, and GPx. ML-385 pretreatment reversed the above changes in the palmitoylcarnitine group, suggesting that palmitoylcarnitine alleviates I/R-induced hepatic injury by activating the Nrf2/HO-1 pathway. The above results suggest that the protective effect of *L. reuteri* against hepatic I/R injury may be related to promoting elevated levels of palmitoylcarnitine. JNK/p38, ERK, and NF-κB signaling activation are strongly associated with hepatic I/R injury [20–22]. In the present study, we found that *L. reuteri* and palmitoylcarnitine reverse the I/R-induced significant increase in the levels of p-p65/p65, p-IκB/IκB, p-p38/p38, p-JNK/JNK, and p-ERK/ERK.

*L. reuteri* was able to alter I/R-induced changes in other metabolites (e.g., spinacoside C). However, we did not delve further into the mechanism, which is a limitation of our study. In future studies, we plan to delve deeper into the impact and potential mechanisms of differential metabolites derived from *L. reuteri* on hepatic I/R injury.

## Conclusion

*L. reuteri* exerts a beneficial effect in mitigating hepatic injury caused by I/R. This protective effect is possibly attributed to various reasons, including the enhancement of probiotic enrichment, such as *Bifidobacterium* and *Lachnospiraceae NK4A136*, the maintenance of metabolic balance characterized by differential metabolite homeostasis like palmitoylcarnitine, and the activation of the Nrf2/HO-1 pathway. These findings offer valuable insights into the possible clinical use of probiotics for treating hepatic I/R injury.

### Electronic supplementary material

Below is the link to the electronic supplementary material.

Supplementary Material 1: Table S1 Antibody information for western blot. Table S2 Antibody information for IF staining.

Supplementary Material 2: Additional file 1 Original Images for Blots.

Supplementary Material 3: Figure S1. A schematic figure of the experimental procedures.

Supplementary Material 4: Figure S2. Pearson correlation coefficient analysis (A) Correlation analysis of spinacoside C with differential gut microbiota. (B) Correlation analysis of spinacoside C and ALT, AST, MPO, Nrf2, and HO-1 levels.

Supplementary Material 5: Figure S3. Pearson correlation coefficient analysis (A) Correlation analysis of Cob(I)yrinate a,c diamide with differential gut microbiota. (B) Correlation analysis of Cob(I)yrinate a,c diamide and ALT, AST, MPO, Nrf2, and HO-1 levels.

## Data Availability

The 16S rRNA sequencing data are deposited at https://www.ncbi.nlm.nih.gov/bioproject/PRJNA1036033. The other data that support the findings of this study are available from the corresponding author upon reasonable request.
