# Supplementary figures and images for: Lactobacillus reuteri mitigates hepatic ischemia/reperfusion injury by modulating gut microbiota and metabolism through the Nrf2/HO-1 signaling
Source: Biol Direct. 2024 Mar 18;19:23. doi: 10.1186/s13062-024-00462-5 (PMC10946149; doi:10.1186/s13062-024-00462-5)

Figure 1D

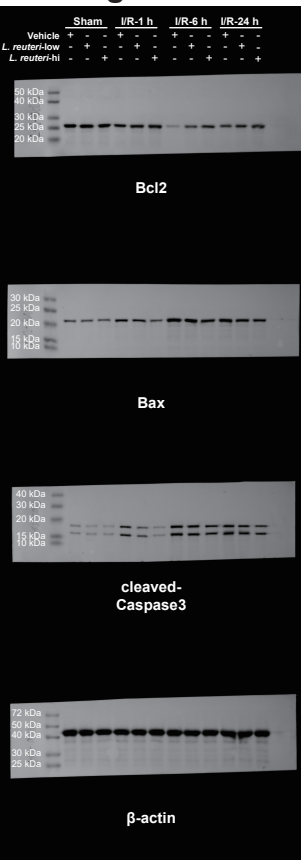

Figure 1E

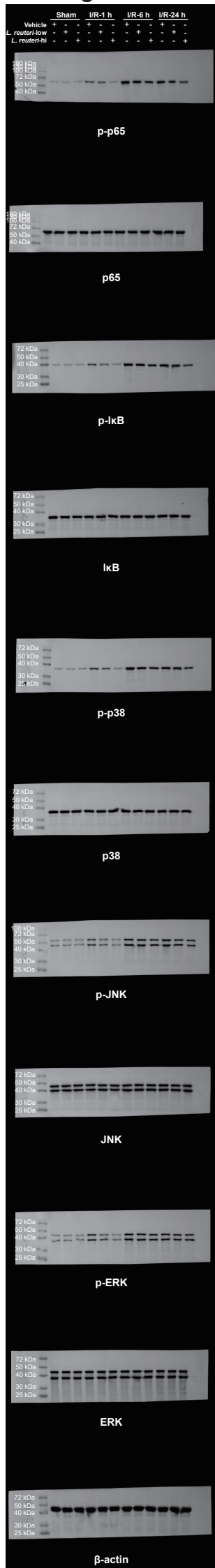

Figure 2C

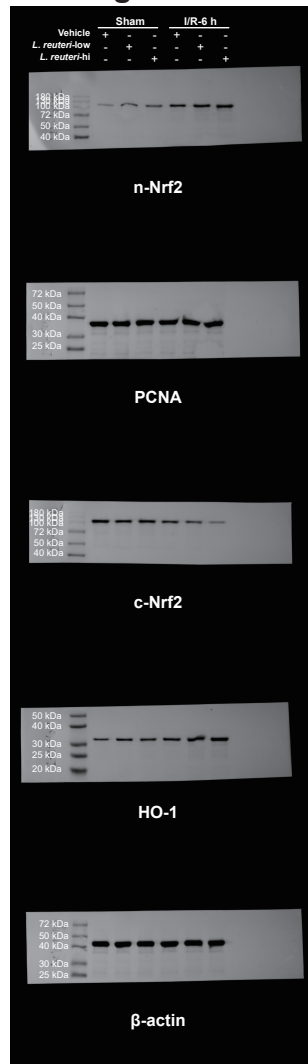

Figure 6D

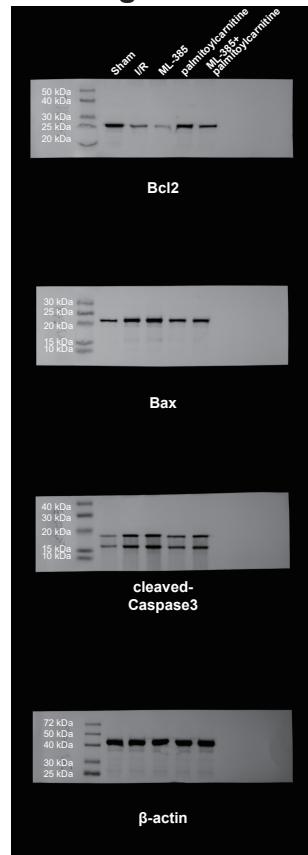

Figure 6E

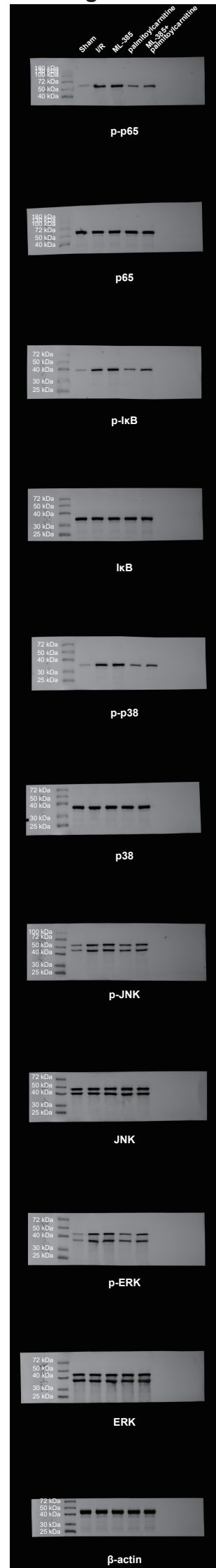

Figure 6H

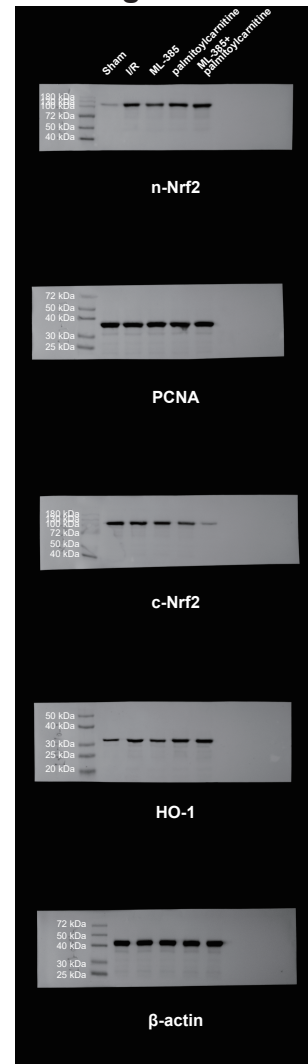

Supplement: Supplementary file 2 — Supplementary Material 2: Additional file 1 Original Images for Blots. [file 13062_2024_462_MOESM2_ESM.pdf]

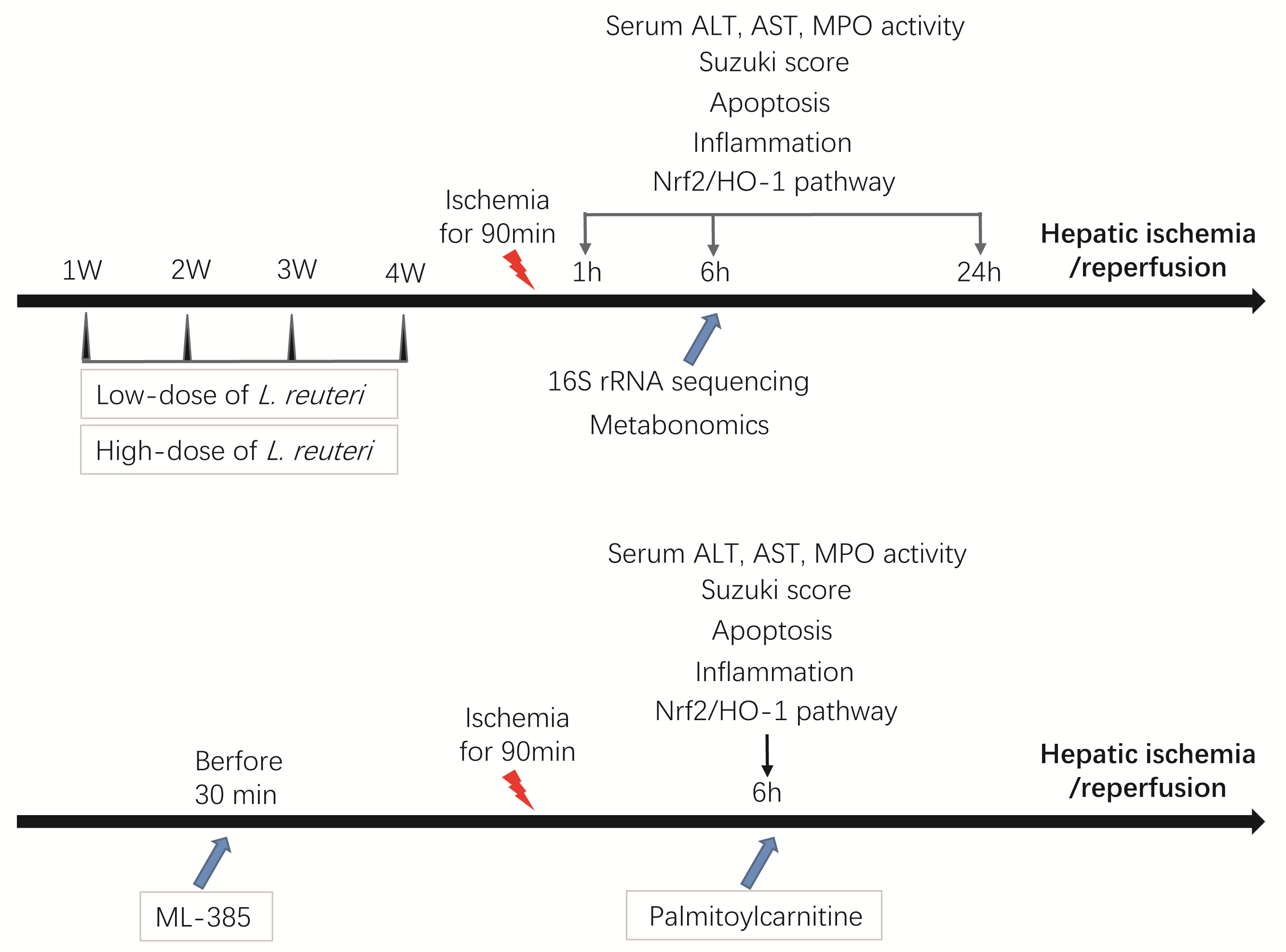

Supplement: Supplementary file 3 — Supplementary Material 3: Figure S1. A schematic figure of the experimental procedures. [file 13062_2024_462_MOESM3_ESM.tif]

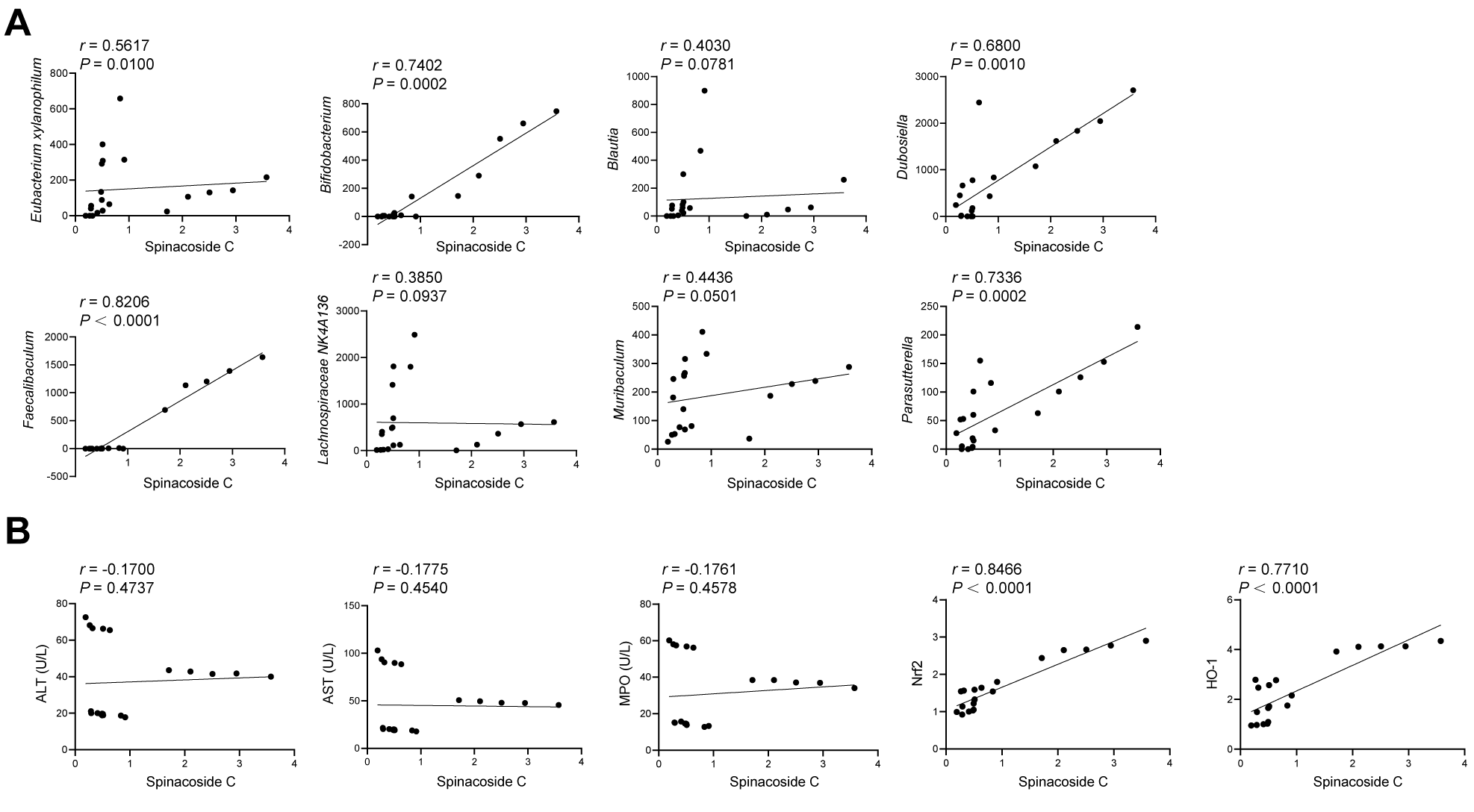

Supplement: Supplementary file 4 — Supplementary Material 4: Figure S2. Pearson correlation coefficient analysis (A) Correlation analysis of spinacoside C with differential gut microbiota. (B) Correlation analysis of spinacoside C and ALT, AST, MPO, Nrf2, and HO-1 levels. [file 13062_2024_462_MOESM4_ESM.tif]

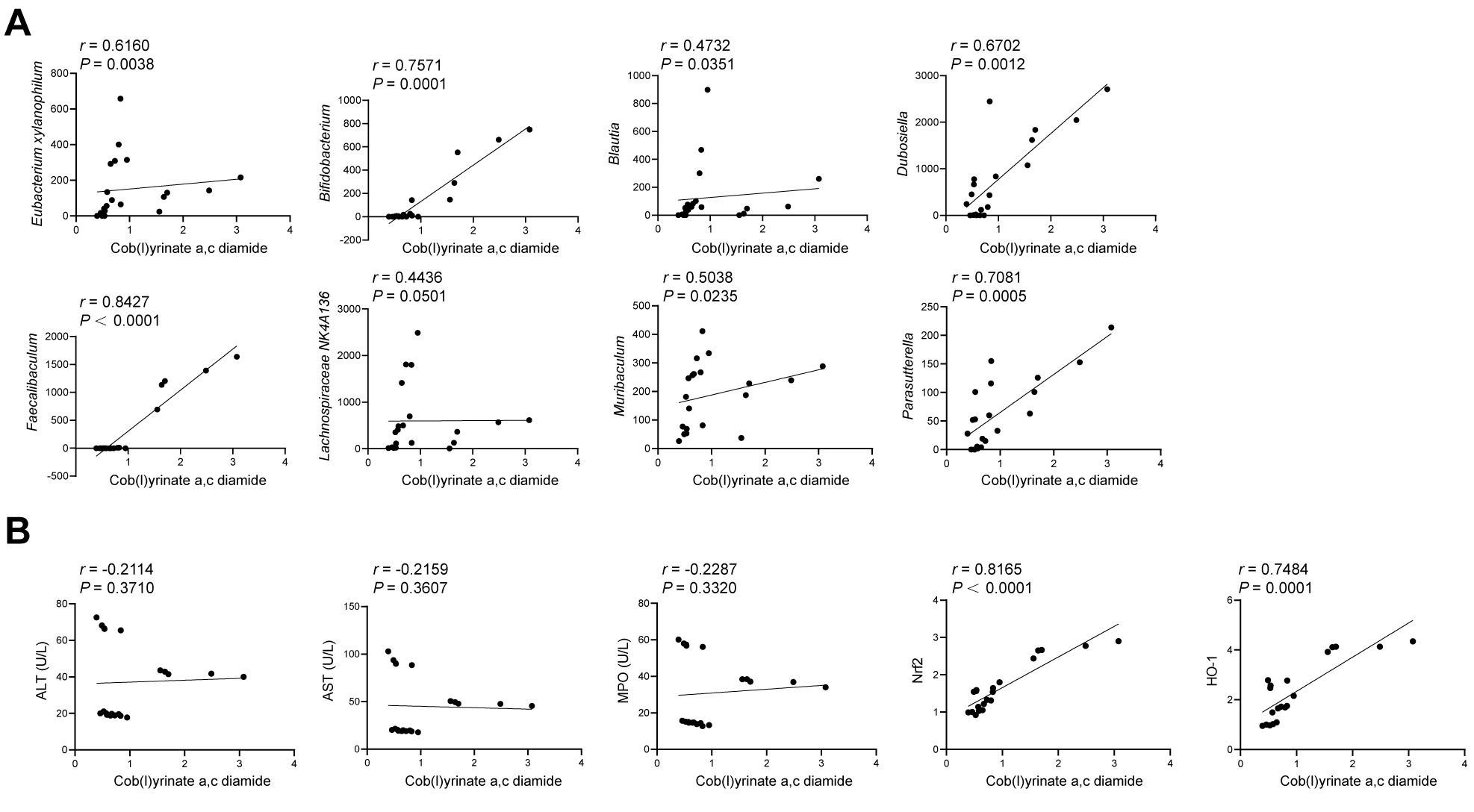

Supplement: Supplementary file 5 — Supplementary Material 5: Figure S3. Pearson correlation coefficient analysis (A) Correlation analysis of Cob(I)yrinate a,c diamide with differential gut microbiota. (B) Correlation analysis of Cob(I)yrinate a,c diamide and ALT, AST, MPO, Nrf2, and HO-1 levels. [file 13062_2024_462_MOESM5_ESM.tif]
